# Impact of Natural Disasters on Mental Health: Evidence and Implications

**DOI:** 10.3390/healthcare12181812

**Published:** 2024-09-10

**Authors:** Eamin Z. Heanoy, Norman R. Brown

**Affiliations:** Department of Psychology, University of Alberta, Edmonton, AB T6G 2E9, Canada; nrbrown@ualberta.ca

**Keywords:** transition, disaster, depression, anxiety, PTSD, mental health

## Abstract

Natural disasters are large-scale catastrophic events, and they are increasing in frequency and severity. Converging evidence indicates that the mental health consequences of disasters are extensive and are often associated with trauma and the disruption of personal and socioeconomic factors in people’s lives. Although most individuals experiencing disaster-related traumatic events do not develop mental illnesses, some experience adverse psychological effects of disasters. These mental health effects begin immediately following a disaster and may persist for extended periods. In this article, we summarize the literature findings to provide a narrative review that focuses on the mental health consequences of natural disasters. An overview of the disaster mental health research field is provided, and the findings are ordered into theoretical frameworks. Then, the development and course of psychopathology regarding disaster aftermath are described in a methodological context. Next, understanding a disaster as an event of transition is highlighted, and the impact of this disaster-specific transition is discussed. Lastly, a potential relationship between the transitional impact of a disaster and mental health consequences is speculated on, and the implications are discussed. The impact of disasters on mental health can be direct or indirect, short-term or long-term, and to some extent depends on the recovery process of the affected community. Also, we propose the possible merits of using the Transitional Impact Scale in the context of disaster mental health research by assessing the features of disaster-related transition and its effects on mental health. We conclude by suggesting a direction for future research in terms of measuring the disaster mental health effects in community settings (affected vs. non-affected) and also considering cross-cultural and cross-regional differences. In recent decades, a large amount of knowledge has been gathered from disaster mental health research, but, still, more research is needed to resolve some irregular findings through refining the methodological variations.

## 1. Introduction

In broad terms, a disaster is a diverse set of events ranging from man-made (e.g., terrorism) to nature-caused (e.g., a tsunami) [1]. These are unexpected and uncontrollable events that have a major impact on people’s personal, social, economic, and well-being aspects of their lives [2,3,4]. By definition, natural disasters are sudden and extreme weather events that can unfold within a brief amount of time, e.g., from minutes to hours (earthquakes or cyclones) or slow-paced events that can last days, weeks, or months (floods or wildfires) [5]. The frequency and severity of natural disasters have increased over recent decades, and there has been a growing concern that they will continue to increase in the coming years [6,7,8,9,10]. In 2020, approximately 100 million people were affected by disaster events, and these events caused an estimated USD 190 billion in global economic losses [11,12].

According to McFarlane and Norris [13], natural disasters are also stressful events that have an acute onset, are experienced collectively, and are time-delimited. Nearly one-third of disaster-affected people may experience a negative mental health consequence such as post-traumatic stress disorder (PTSD), anxiety, depression, and others [14,15,16]. Therefore, there has been growing recognition of the importance of mental health assessment in disaster response strategies [1,17]. This is because, although, following a disaster, most people cope well [5,18], not everyone continues to function normally; some affected individuals experience trauma related to disaster exposure, while others experience delayed onset of psychological impairments, and a small fraction of these develop more serious mental health issues in the long run, e.g., PTSD [14,19,20]. Thus, the disaster impact can extend beyond its onset and last for extended periods [21,22,23,24].

Additionally, a significant amount of work has recognized that, intersecting with disaster exposure, several social and environmental factors (e.g., displacement and change in close relationships) and daily-life-related factors, such as low income, loss of job and properties, loss of family members, etc., and financial instability have the potential to cause or exacerbate negative psychological effects [25,26,27,28,29,30,31]. In other words, these natural calamities can have both short- and long-term adverse effects on mental well-being as a consequence of changes owing to socioeconomic conditions, social disruption, displacement, and financial loss [16,20,32].

Several recent scoping and narrative reviews have explained the various ways disasters can negatively impact the affected populations, both in the short term and long term [33,34,35,36,37]. However, they did not clarify how disasters catalyzed material and psychological changes that affect people’s lives (i.e., the nature and extent of these changes) or how these changes differ between affected individuals. That being said, there is a growing need in disaster mental health research to develop an understanding of the aspects of disaster-related changes or transition (i.e., pre-disaster to post-disaster) and the impact, and the link between this transitional impact of a disaster and mental health. In this paper, we attempt to address this shortcoming by considering why some people are more vulnerable to being negatively affected by a disaster while others are relatively unaffected by the same disaster event.

Therefore, building upon those recent reviews, the current narrative review aims to assess the existing disaster-related mental health research and explore the key literature gaps related to the impact of natural disasters on mental health from the perspective of transition: pre-disaster to post-disaster. To achieve this aim, first, we provide an overview of some of the classic theoretical models and frameworks with supporting study findings. Then, we provide a general description of the course of mental health issues following disasters with some research evidence that includes different methodologies. Next, we develop the notion that disasters can be understood as potentially important transitional events and discuss the nature and extent of the impact of this disaster-specific transition. Then, we consider the possible relationship between disaster transitional impact and mental health and discuss its implications. Finally, we conclude by suggesting the future direction of disaster mental health research.

### Search Strategy and Screening Process

The review was conducted through a general search of the literature on the following search engines: PsycINFO, PubMed, Web of Science, and Google Scholar (the first ten pages of the search results). The search was conducted using natural disasters and mental-health-related terms to identify the relevant literature. For example, the search terms included disaster, natural disaster, flood, earthquake, wildfire, cyclone, tornado, hurricane, drought, mental health, mental illness, well-being, psychological distress, depression, anxiety, and PTSD/post-traumatic stress disorder.

All the papers published until April 2024 and available in the above-mentioned search engines were reviewed. The titles and abstracts of the published articles were screened for eligibility, that is, the relevance to the question of how natural disasters can affect mental health. The key findings in the literature were summarized by author, year, methods, and results, and the procured information was qualitatively synthesized to inform the current narrative review.

## 2. Theoretical Frameworks/Models of Disaster–Mental Health Relationship

In the past few decades, several frameworks and models have been developed in efforts to explain the multiple influences of a disaster that affects a person’s well-being [38,39,40,41]. Although the frameworks differ in their details, there is converging evidence that well-being not only depends on personal factors (e.g., pre-existing mental condition, lifestyle, behavior, and attitude) but also on socio-environmental factors [42,43,44,45]. These factors interact with each other and together have an impact on people’s mental health [36,46].

Generally speaking, these models and frameworks can be classified into two broad categories: stress–reaction-focused and psychosocial-focused. Stress–reaction models focus on the distress-specific psychological outcomes of experiencing a disaster, particularly depression, anxiety, and PTSD [23,24,47,48]. In contrast, the focus of the psychosocial models is on the role of social and environmental factors on mental health, and the ways disasters disrupt these factors and thus give rise to adverse well-being consequences [36,40,49]. Note that these two broad frameworks are not standalone concepts; rather, they overlap because of the interactive nature of social, environmental, and individual factors. Recognizing this limitation, here, these models are described taking into consideration the interconnected quality of socio-environmental and personal determinants to meaningfully structure the wide-range impact of natural disasters on well-being.

Below, we describe the existing theoretical frameworks of disaster mental health effects from either a stress–reaction context or psychosocial context. We also describe another model type that is less empirical and more practical that focuses on the psychosocial recovery process of the well-being of the disaster-affected community as a whole.

### 2.1. Stress–Reaction-Focused Frameworks

The overall notion of these frameworks is that natural disasters can directly affect mental health by exposing people to psychological injury [50,51]. The most extensively studied psychological issue is PTSD because it is usually the most prevalent disaster-related mental health outcome [3,40,52,53,54,55]. Thus, most of the stress–reaction frameworks have been developed while maintaining their focus on PTSD [15,49]. In one of the earliest works, Green and colleagues [56] proposed a PTSD model that describes the mental outcomes of disaster survivors considering disasters as traumatic events [56,57]. According to this model, disaster exposure leads to mental processing of the event involving intrusion and re-experiencing phenomena that provoke avoidance and denial responses. This processing takes place in the context of various personal (e.g., previous psychological issues and coping style) and social (e.g., social support and socioeconomic status) factors. These factors together determine whether an individual can successfully process the disaster event and the nature of the psychological outcome, i.e., PTSD.

In support of this model, Green and Lindy performed a literature review of studies on different disaster types, such as volcanic eruptions, floods, and landslides, and their impact on mental well-being [57]. The findings of the review indicate that PTSD is the most common diagnosis associated with a disaster, and the post-disaster PTSD rates were higher (11% to 22% depending on the exposure type and duration) in the disaster-exposed samples, whereas a comparison (unexposed) sample had a rate of 8% from other causes, e.g., accident or crime [58,59,60,61,62,63]. Interestingly, they also found the PTSD rates to decline over time. For example, the samples collected following the Mt. St. Helens volcanic eruption, Puerto Rico floods, and mudslide had only a 4% rate of disaster-related PTSD in the two years following the disaster, and, for the most part, it abated by the third year [59,60]. However, although the symptoms decreased over time, the levels did not return to normal, and many individuals were still affected many years after the precipitating disaster [64]. One caveat is that the model is more appropriate for explaining the long-term impact of a disaster because most of the relevant studies focused on the first 12 months to the following years of the disaster event, missing the short-term or immediate effects of the disaster.

In 2012, North and colleagues [65] proposed another natural-disaster-related PTSD model. Unlike Green and colleagues’ model [56], this model was comprehensive in the sense that it explained PTSD as a straightforward psychological consequence of disaster events rather than from the perspective of disasters being traumatic events per se. In this model, the factors associated with the mental health sequelae of disasters included personal traits, disaster severity, degree of disaster exposure, disaster community characteristics, and other negative life events. In their systematic review [65] of the combined data from 811 survivors of 10 disasters between 1987 and 1995, they found that PTSD was the most prevalent mental outcome of the disasters, and the risk of developing PTSD following a disaster is associated with the severity and greater exposure to the disaster, female sex, younger age, ethnic minorities, lower socioeconomic status and education, marital status (married women and unmarried men), other stressful life events, pre-disaster psychiatric illness, disaster injury or witnessing death, and lack of perceived and actual social support. They also found that intrusion and hyperarousal symptoms of PTSD were prevalent among most of the survivors, and avoidance or numbing symptoms were a significant marker for PTSD onset [52,66]. Although this model was better designed in terms of using consistent research methods (uniform assessment and timeframe), it lacks a clear distinction between the short- and long-term PTSD outcomes of a disaster due to variability in the timing of assessment (i.e., the amount of time elapsed from the disaster to the assessment).

Later, given the complex nature of natural disasters and their negative effects, a broader definition of psychological distress (e.g., fear, sadness, and hopelessness) was adopted by disaster mental health researchers [67,68]. In a more recent work, Saeed and Gargano [37] outlined a stress-mediated framework that highlighted two important concepts. First, everyone reacts differently to the same distressful situation; second, distress in an individual can be adaptive and manageable, or maladaptive. They also highlighted the fact that extreme fear and uncertainty are common reactions to natural disasters and that these emotions can contribute to an elevated stress response. These authors argue that a distress situation like a disaster event can give rise to a five-step stress response. First, the distressful situation causes fear and worry about one’s own health, financial or employment situation, or loss of reliable support services and the well-being of close others; second, the distress causes symptomatic stress responses such as changes in eating pattern, sleep difficulty, use of a substance, etc., which do not fall under diagnostic criteria of psychological issues; third, the distress situation may facilitate an aggravated onset of an existing mental problem that was stable until exposure to disaster; fourth, it may cause a first-time episode of a psychological issue in those who are already predisposed to biological or genetic vulnerability; finally, it may cause stress-related problems such as anxiety disorder, PTSD, depression, or an adjustment disorder among disaster-exposed individuals.

**Figure 1:**
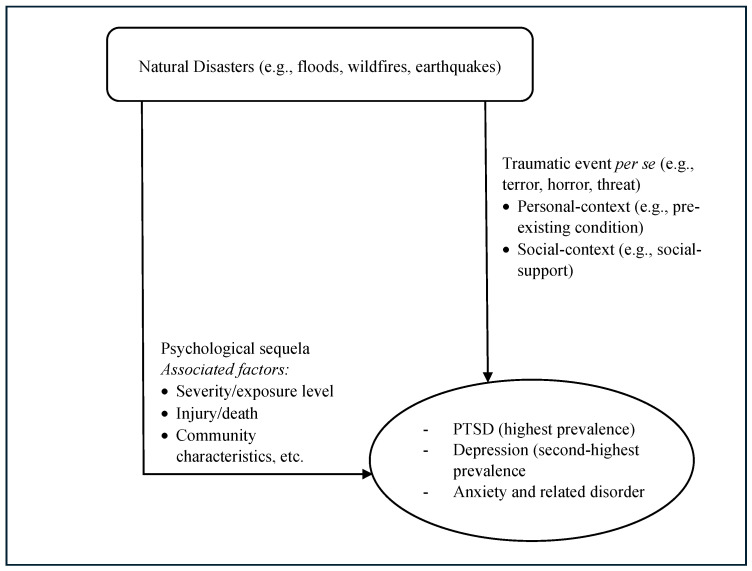
Stress–reaction-focused framework outline.

In their literature review [37], they found that experiencing a disaster event increases the prevalence of a range of mental problems, such as PTSD, depressive disorder, anxiety, grief, and loss, etc., especially in the directly disaster-exposed group and in individuals who have lost support and resources. The highest prevalence rate was for PTSD (34.4%), followed by depression (25%). The severity of trauma and psychological distress were also found to be major factors in the post-disaster mental outcomes. In addition, a prior history of stress and pre-existing mental conditions are vulnerability factors causing an increased risk of developing psychological problems in the disaster aftermath. Moreover, a gene-by-environment interaction was found in individuals predisposed to biological and genetic factors, causing a higher likelihood of mental illness. This is one of the frameworks that underlines the importance of pre-existing psychological conditions and the growing area of work on genetic variables related to risk and protective factors in developing mental health problems in the context of natural disaster impact. However, it is not clear whether this distress-mediated pathway can describe both the short-term and long-term impacts of a disaster on mental health. Figure 1 depicts a general outline of the stress–reaction-focused framework.

### 2.2. Psychosocial Frameworks

Disaster mental health researchers have long followed the frameworks used in the field of stress studies and the methodological features of sociology [38,39,42,69,70,71]. The early heuristic model of the stress process indicates the indirect influence of a discreet stressful event on well-being, where environmental stressors (e.g., traumatic event, daily hassle, and chronic condition) create demands for biological (e.g., physiological response) and psychological (e.g., subjective perception) changes. These changes put people at risk of developing physical- and mental-health-related issues [69,72]. Later, psychosocial factors such as social support and coping were also found to be useful in assessing the individual differences in how stress affects well-being [73]. Disaster mental health researchers adapted this psychosocial concept of stress to test the relationship between disaster exposure and the associated mental health outcomes [71].

One of the earliest psychosocial models was the Conservation of Resources (CORs) model, which is based on the relationship between the psychosocial factors and the psychological outcome [74]. This model proposes that the loss or threatened loss of social (e.g., family roles and work roles) and personal (e.g., optimism) resources results in reducing the capacity to cope and provokes psychological distress. Further, the model implies that, by “reloading” diminished resources, coping capacity can be enhanced, and psychological distress can be reduced [74,75]. In a disaster context, the CORs model implies that, following a natural disaster, the coping options are restricted and psychological distress is produced when there is a loss of resources, such as object resources (house, vehicle, etc.), condition resources (community network, employment, etc.), personal factor resources (sense of purpose, sense of self, etc.), and energy resources (time, money, information, etc.) [39,49,74,76]. Moreover, in post-disaster conditions, if targeted aids are provided to these affected resources, the coping capacity should be increased, which will lower the psychological distress of the disaster victims [74,75,76].

In support of this COR model, in a cross-sectional study [76] with 418 individuals of Hurricane Hugo victims, the researchers found that loss of resources was a significant predictor determining post-disaster psychological distress, with the severity of resource loss being associated with an increased prevalence of mental health problems such as PTSD, anxiety disorders, and depression. In the disaster well-being literature, this model is important in the sense that it considers a range of adverse life changes (e.g., loss of house, financial adversity, and disrupted social support) on individual adjustment following a disaster event, shifting the emphasis from a more direct effect of disaster exposure such as threat, terror, or horror [76,77]. For instance, many natural disaster victims, particularly when provided adequate warning, might not be directly exposed to life-threatening or other horrific disaster-related experiences. However, most disaster victims may face a range of adversities during the post-disaster period, including food scarcity, damage to property, loss of employment, feelings of hopelessness, and disruption of normal routines [78]. Therefore, it is important to consider individual differences in resource availability and vulnerability to the loss of that resource to understand the risk of experiencing post-disaster mental distress.

Despite these strengths, the model is not without its limitations. The model investigates disaster–mental health relationships using only one psychosocial factor, i.e., resource loss, as an overarching term, overlooking the differences within resource types. Also, the model fails to distinguish between pre-disaster availability of resources (e.g., social support and financial support) and post-disaster vulnerability to resource loss (e.g., loss of belongings and loss of employment) [49]. For example, certain groups of people (e.g., single parents and unemployed people) might already have fewer pre-disaster resources and thus be more vulnerable to resource loss, coping impairment, and psychological issues during the post-disaster period, making them susceptible to developing mental problems like depression, anxiety, and stress-related disorder. In addition, the model more appropriately explains short-term adjustment than long-term adjustment [76].

A more recent psychosocial framework addresses the ways a disaster affects the social and environmental determinants of mental well-being. Inspired by Dahlgren and Whitehead’s social equity model in health [79], Lawrance and colleagues stated that [36] a person’s mental health and well-being are influenced by various interacting psychosocial factors that reside in one’s society and environment. They categorized these factors or determinants into nested layers: socioeconomic, cultural, and environmental factors are in the outermost layer, which includes consecutive layers ranging from living and working conditions, social and community networks, individual psychology and lifestyle factors, and demographics and personal traits. The layers specified in the model and the psychosocial factors that are part of each layer are connected and interactive. In this view, disruption at one layer has implications for all the other layers. For instance, a person’s demographics and factors such as pre-existing mental conditions interact with their attitude, behavior, and lifestyle. This influences their wider social and community networks. All these layers reside within the layer of their living and working conditions, that is, whether they have access to shelter, food, and other basic needs, and have stable livelihoods including income. The outmost layer, socio-economic and environmental conditions (e.g., financial, political views, and culture) interacts with these other layers nested within it.

In a disaster well-being context, the effects in one layer will have an impact across the other layers, ultimately affecting the mental health and well-being of individuals. In their literature review [36], Lawrance and colleagues provided a comprehensive overview of how disaster events interact with the mental health of disaster-affected people. They found disaster to disrupt socioenvironmental and individual conditions, leading to a range of outcomes such as injury, death, loss or damage to property, changes in living, and evacuation, which can lead to an increased risk of developing post-disaster mental problems such as PTSD, anxiety, and depression among the affected population.

**Figure 2:**
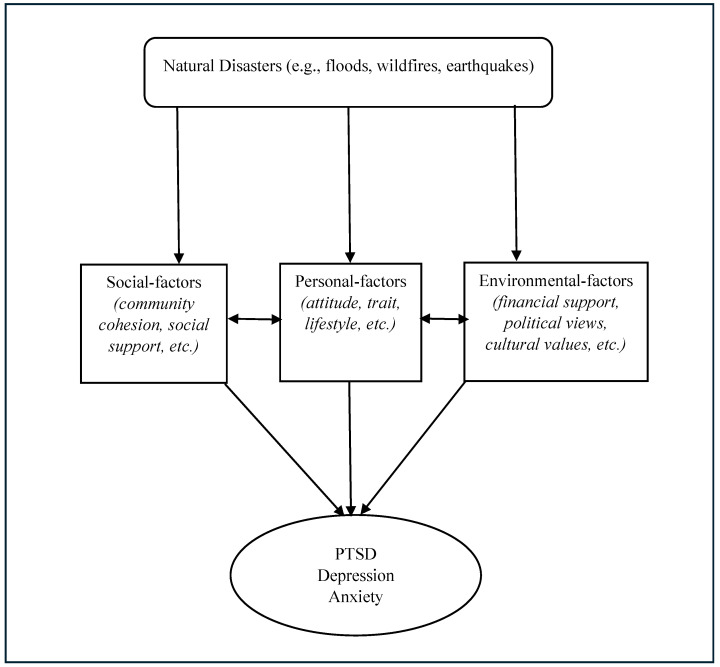
Psychosocial framework outline.

In the context of a disaster, the importance of the model is that it highlights a set of consecutive changes. In other words, it specifies how all the layers and determinants change over time as a result of changes in the life stages of an individual and the global and personal environment they live in. Although this model highlights the importance of the interactive and dynamic influence of psychosocial factors on disaster mental health, the interaction and influence of the multiple factors are complex and context-dependent. Figure 2 outlines the general concept of the psychosocial framework.

### 2.3. The Direct and Indirect Pathway

**Figure 3:**
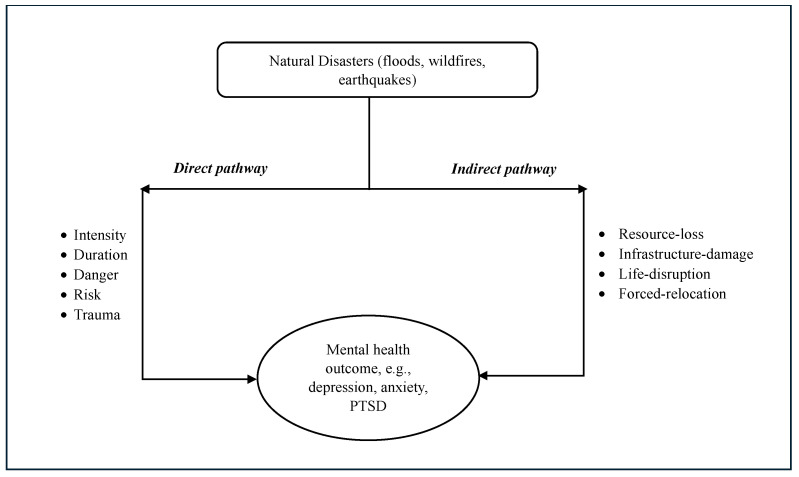
Pathways linking disasters and mental health.

Some disaster mental health researchers have conceptualized these stress–reaction and psychosocial models of disaster well-being relationship as direct and indirect pathways, through which a natural disaster affects people’s mental health and well-being [35,36,40,80,81,82]. To illustrate, a disaster may directly affect mental health by exposing individuals to psychological trauma related to its intensity and duration, whereas indirect effects may occur through injury, death, loss, disruption to life, and damage to personal property and social infrastructure as a consequence of a disaster [35,36,40,82]. Notably, these two concepts are not distinct; rather, they are parts of a continuum [36,40]. This continuum approach, from direct to indirect, is appropriate in highlighting the wide-range impact of disasters on mental health (Figure 3). Some researchers adopted this continuum approach for simplicity and to meaningfully structure these impacts [40]. For example, the findings from several reviews [35,36,81,82] indicated that, on one hand, adverse weather events such as floods and wildfires may expose people to risk and physical danger, which cause increased risks of depression, anxiety, and PTSD (direct pathway); on the other hand, these disaster events may also cause loss of home and job, damage to infrastructure, forced relocation, and disruption to social and community resources, which can lead to elevated rates of anxiety and other adverse mental outcomes (indirect pathway).

### 2.4. Community Recovery Models

In the last decade, apart from the aforementioned stress–reaction and psychosocial models, community-based post-disaster recovery has garnered considerable attention from disaster mental health researchers. Models have been developed that report on how disasters affect community well-being as well as individual mental health [4,82,83,84]. These models are stressor-based and focus on the collective emotional reaction and recovery process of the affected community [4,43,84,85]. Also, according to these models, the affected communities often progress through various phases of psychosocial recovery, and, notably, these phases have been articulated less by empirical studies and more so by the field experience of disaster mental health experts [1,5]. These phases of disaster effects are particularly relevant from the perspectives of disaster management planning, policy development, and program implementation as they provide an understanding of community response and recovery and the relationship between community-level resources and post-disaster well-being risk [4,5,53]. Although these recovery models incorporate elements of psychosocial frameworks, they also differ in the sense that they primarily focus on recuperating the affected ‘community’ (individuals are nested within), involve multiple agencies and stakeholders, and have multiple priorities evolving over time [5,84]. The outline of these models involves multiple phases of disaster impact on the community’s well-being.

Tyhurst was one of the first to suggest phases of disaster impacts [86]; the first is the *recoil phase*, in which the initial distress is replaced by a sense of self-consciousness. This is followed by the *post-impact phase*, during which disaster survivors have to deal with the disaster experience. Finally, there is the initial *recovery phase*. This basic model primarily explains individual reactions to a disaster and is unclear about the overall response to the distress as a disaster-affected community. Later, the Adaptation and Development after Persecution and Trauma (ADAPT) model was proposed by Silove, which aimed at linking various dynamic psychosocial factors to a wide range of mental health issues of populations exposed to stressful events like disasters [83]. This model suggests that the impact of stressful events can be arranged by the effect level on five inter-related psychosocial domains that, during normal circumstances, stabilize societies. These domains are (i) Safety/Security; (ii) Bonds/Networks; (iii) Justice; (iv) Roles and Identities; and (v) Existential Meaning. Rebuilding these domains is regarded as important in restoring community mental health and psychosocial recovery in disaster aftermath. Although this model can potentially be informative regarding the types of interventions needed to obtain community recovery, many of the psychosocial domains, for example, existential meaning, might include abstract concepts that can be a challenge in grasping the full meaning.

Subsequently, to minimize the short- and-long term effects of disaster events, and to consider disaster planning and resource allocation [5,84], an effective and extensive model was proposed by the Federal Emergency Management Agency (FEMA) [87,88]. This model was intended to detail the collective emotional reaction and recovery process of the affected community and to assist in related policymaking and disaster recovery program development.

**Figure 4:**
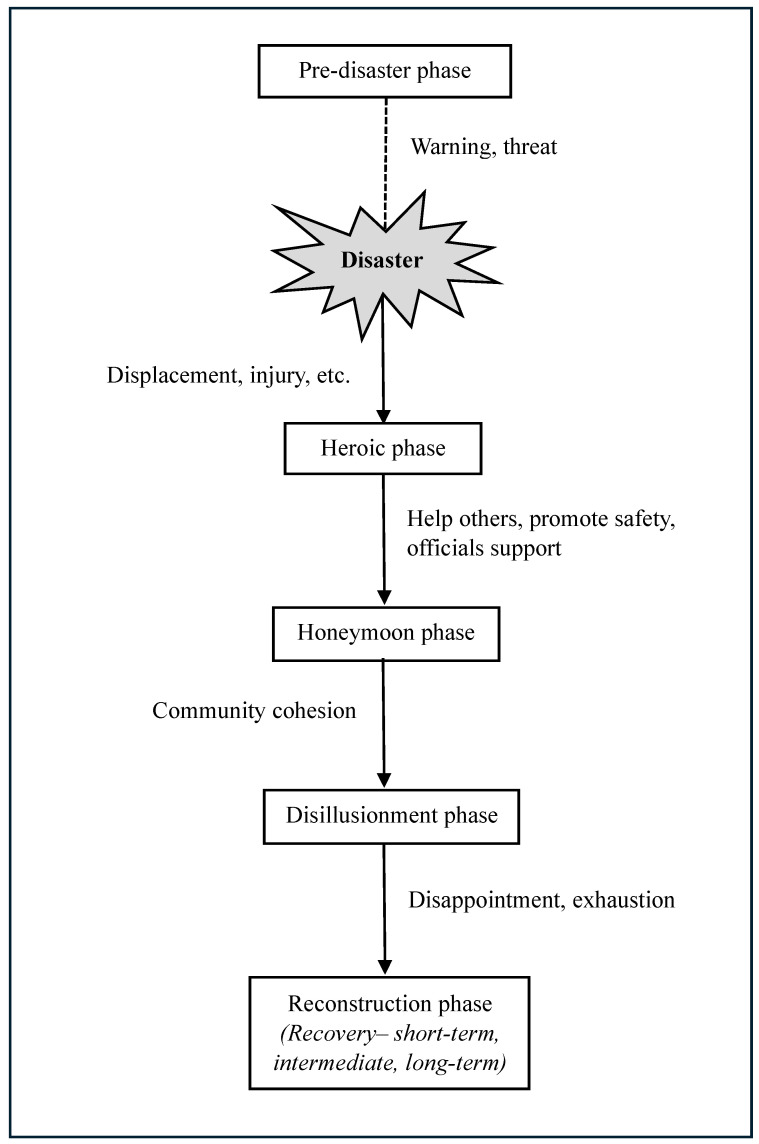
Phases of community recovery process.

This FEMA model suggests that the disaster-affected community often progresses to recovery through six phases (Figure 4). The *pre-disaster phase* includes warnings and threats related to the looming disaster. During the *disaster impact phase* (at the outset), various sub-events (e.g., displacement and injury) occur, and emphasis is placed on exerting control over what is occurring. After that, the *heroic response phase* involves saving others, temporary relocation, promoting safety, and support from government and insurance agencies. Following that, there is a *honeymoon phase* where external assistance remains and community bonding increases through a shared catastrophic experience. Survivors’ level of emotion remains high, that is, being hopeful, and believing that the help they receive will make them whole again. The honeymoon phase may occur up to several months after the disaster, but the actual timing and length of this phase may vary. The honeymoon phase is followed by a *disillusionment phase* characterized by disappointment. Disaster survivors are disillusioned because agencies and volunteers pull out their assistance and the community’s hopes for restoration to pre-disaster wellness are typically unfulfilled. During this phase, community cohesion is weakened as individuals focus more on their unmet needs. The least impacted neighborhoods return to life as usual, which can discourage and alienate more people living in more severely impacted neighborhoods. Survivors may become exhausted due to growing multiple demands, including financial pressures, relocation or living in a damaged home, family discord, bureaucratic hassles, and a lack of free time for recreation or self-care. Mental problems and the exacerbation of pre-existing conditions emerge as a result of ongoing stress and fatigue. The disaster anniversary experience occurs during this phase, and failure to address this experience can further demoralize survivors, exacerbate underlying psychological distress, and worsen the trajectory of community recovery. Finally, the *reconstruction phase*—including short-, intermediate-, and long-term recovery—begins a few days after the disaster but can last up to several years depending upon the level of the disastrous effect. Survivors attempt to restore their lives by rebuilding homes, returning to old jobs, finding new employment, and resuming or forming new social support systems. Some can accept new circumstances, including losses and changes, whereas others may instead focus on resentment and anger, identifying themselves as victims.

Note that individuals progress through these phases at different times. Consequently, in response to the same disaster event, people manifest different psychological symptoms over different timelines. Moreover, depending on the severity of the experience, availability of resources during and after the event, and individual factors, many people will develop persistent symptoms over the years. For example, when community members of Slave Lake, Alberta, Canada, who experienced a devastating wildfire in 2011, were surveyed one year after the fire [4], they exhibited low levels of recovery and cohesion—findings that corroborate the FEMA model expectations for a community in the disillusionment phase of recovery. This finding not only suggests empirical support for the phases of disaster proposed by FEMA but also suggests that the model may have implications for disaster recovery program planning [84].

It should also be noted that this psychosocial model of community recovery was developed within the context of disasters occurring in North America and may have the greatest applicability to developed countries [4,5]. For example, the FEMA model was implemented in the recovery services for the 2013 flood victims in Southern Alberta [4]. Although this model is unique in the sense that it helps to understand both community response and individual response (within that community), the generalizability is limited (i.e., specific to developed nations) and does not consider the quality of post-disaster changes that might vary across communities, causing different outcomes. For example, an annual flood in Morris, Manitoba, might not produce highly negative emotional symptoms, whereas an unprecedented wildfire in La Ronge, Saskatchewan, may well give rise to adverse emotional reactions [4].

In summary, the link between disaster exposure and mental health impact can be broadly understood on three levels. First, the frequency of disaster events is escalating, and, hence, disasters are affecting various aspects of the lives of an increasing number of people. Second, the nature and extent of disaster exposure are associated with a broad range of mental health problems, e.g., depression, anxiety, and PTSD. Third, although psychological distress can dissipate with time, difficulties with adjustment often persist following disaster exposure.

Again, numerous studies have been conducted to assess the impact levels of disasters on mental health based on those aforementioned theoretical models. In those studies, various methods of research were used to evaluate both the short-term and long-term mental health consequences in disaster aftermath. In the following section, we describe the differing trends and prevalence of mental health problems after disasters in the context of different research methods.

## 3. The Course of Mental Health Issues Following Disasters

Natural disaster mental health is an emerging field of research. One of the earliest studies in this literature concerned an earthquake in Macedonia during the 1960s, reporting that people were mildly distressed and that severe mental disturbances were relatively uncommon during the first few days after the earthquake [89]. Previously, disaster research had primarily focused on directly exposed groups that were physically injured or endangered because of the disaster trauma experience [52]. Later, in the 2000s, disaster mental health researchers became interested in the psychological consequences of those groups that were not directly exposed to the disaster but were impacted in some way, e.g., not living in the disaster region but hearing news about the disaster [90]. The focus was to measure the scope and magnitude of the disaster on large populations across wide geographic areas, providing opportunities to explore the differential effect of disaster exposure type (direct vs. indirect) and their mental health consequences [52].

To date, the research on mental health sequelae of disasters has produced considerable data on the prevalence of psychological responses to disasters. These studies have found that the mental health consequences differ between impacted individuals and between affected communities, from disaster to disaster, and from one time frame to another [52]. We already know that, following a disaster, the majority of people do not develop psychological issues and cope well, recovering to their previous level of functioning [19,91]. However, it is not that they are completely unaffected; rather, they adapt to situational demands and remain effective in their work and family life. In contrast, some of the affected individuals experience adverse mental health effects [3]. The psychological impact of disaster begins immediately after the event and may persist for months and sometimes even years [5]. As stated earlier, mental problems that are most likely to develop after a disaster are PTSD, depression, and anxiety [92]. Note that these psychological issues rarely occur in isolation and have a highly comorbid nature among them [53,93]. In fact, this psychopathological comorbidity is often considered normal for individuals with PTSD [94,95]. Chiu and colleagues provided a potential explanation for this comorbidity; these disorders are psychological consequences or reactions of traumatic event exposure (e.g., disaster), and the overlapping symptoms among these disorders account for their co-occurrence [96].

### 3.1. Design and Methods in Disaster Mental Health Research

The methods of the earliest disaster studies were largely based on observation, anecdote, and clinical interviews combined with symptom questionnaires or psychological tests [52]. An important advancement in the field was the introduction of PTSD diagnosis in the third edition of the diagnostic and statistical manual of mental disorders (DSM-III) in 1980 [3]. In the 1980s and 1990s, several well-designed studies were conducted that provided the first extensive data on the prevalence of PTSD and other psychological problems among disaster-exposed populations [14]. At the same time, two types of assessment techniques started to be widely used for trauma exposure and PTSD: structured diagnostic interviews and self-report measures [97]. In the 2000s, there was a methodological revision, shifting from full, rigorous diagnostic assessment to quick, short self-report symptom screening scales, and the application of rapid sampling methods (e.g., random digit dialing) and web-based data collection [98]. These changes reflected the broader trends of an era in disaster mental health research. In a recent methodological review, Wolbers and colleagues reported that [99], during the first decade (2001–2010), interview, document analysis, and survey methodologies were evenly used in disaster well-being research. However, increased use of the interview method was visible in the second decade (2011–2020), while the use of the survey method remained relatively steady throughout the years.

In terms of study design, the majority of the disaster mental health research was cross-sectional [49,71]. In an early review of the methodological trends across 225 disaster mental health studies, Norris and Elrod reported that 72% of the samples were studied cross-sectionally, and the remaining ones were assessed two times [71]. In more recent reviews, cross-sectional or repeated cross-sectional was found to be the most commonly used design in disaster mental health studies, while a small portion of the studies used longitudinal design [34,100].

For simplicity’s sake, here, we took a design-based approach, focusing only on cross-sectional and longitudinal study designs to provide some evidence on the prevalence of mental health problems following disasters.

#### 3.1.1. Cross-Sectional

As mentioned before, PTSD is regarded as the signature psychopathology of disaster mental health consequences [3,52,101,102]. The reported PTSD rates in cross-sectional studies of different disaster types have ranged from 10% to as high as 50% [14,103,104,105,106,107], although the investigated time varied from 1 month to 37 months after the disaster [108,109,110,111]. For example, a cross-sectional survey on the 2017 earthquake in Mexico revealed that, after 2 months following the quake, 36.4% indicated symptoms consistent with PTSD, with an increased risk found in women, those who had their home damaged, and individuals with pre-existing mental conditions [112]. Another cross-sectional survey, conducted two years after Hurricane Katrina, found that the estimated prevalence of PTSD in the metro area was 30.3%, while, for the total sample, the estimate was 16.3%; also, hurricane-related stressors such as injury, property loss, and job loss were found to be strongly associated with the development of PTSD [103].

After PTSD, depression is the second-most prevalent and commonly studied mental health problem in disaster populations [3,27,37]. The rate of depression ranged between 5.8% and 54%, whilst the manifestation of depression symptoms was found to be between one month and 48 months after natural disasters [113,114]. Moreover, the psychosocial factors that were associated with increased risk of depression onset were found to be female gender, older adults, unemployment, property loss, forced relocation, low income, and loss of close others [48,68,115,116,117,118]. For instance, a cross-sectional study surveying individuals displaced from their homes following the 2016 wildfires in Fort McMurray found that, 12 months following the event, the prevalence rate of depressive symptoms was around 15% [111]. In another multi-site cross-sectional survey, 8 months after the 2008 Sichuan earthquake, the estimated rate of depression was found to be increased among those survivors who lost their family or relatives (55.8%) compared to those who did not (37.5%) [119].

Although studied less frequently than PTSD or depression, elevated levels of anxiety have been reported among disaster-affected populations [3,37,120]. Due to high comorbidity, the majority of disaster mental health studies assess the anxiety levels among affected people alongside PTSD or depression measurement rather than examining them alone [53,121]. A feeling of uncertainty, which is a central feature of disaster experience (e.g., housing adversity, job insecurity, or insurance-related distress), can lead to increased anxiety levels after a disaster [122]. The post-disaster prevalence rate of anxiety was reported to range from 2.2% to 48%, while the time of assessment varied from 2.5 months to as late as 17 years [117,123,124,125,126]. Furthermore, the research documented a multifaceted relationship between several stressors experienced by disaster-affected and associated anxiety outcomes [127,128]. These factors included property loss, injury, loss of loved ones, economic loss, displacement, lower social support, and pre-existing mental health conditions [23,53,101,129]. A cross-sectional survey with 2016 Fort McMurray wildfire survivors after 6 months found that the overall prevalence rate of general anxiety was 19.8%, and factors such as pre-existing anxiety conditions, loss of home, relocation, and lack of social support contributed to a two to nearly seven times increased likelihood to develop anxiety symptoms [130].

#### 3.1.2. Longitudinal

Even though most of the work in the disaster mental health field has relied on cross-sectional assessments, several longitudinal and sequential cross-sectional studies were conducted in more recent times to document the course of well-being outcome over time [19,107,131,132,133].

The evidence from longitudinal studies suggested that post-disaster symptoms of mental health problems reach their peak in the year following the disaster event and then improve, whereas, in other studies, adverse psychological symptoms persisted for months and years [3,14]. In general, across a wide range of disasters, the findings indicated an improvement in PTSD symptoms following a high-impact disaster (e.g., between the first few months and 12 months or more), but the prevalence rates of depression and anxiety tend to remain stable over time rather than showing attenuation [18,27,130,134,135]. For example, in a longitudinal study, Pietrzak and colleagues examined the course of PTSD, depression, and anxiety following Hurricane Ike at three post-hurricane time points [136]. They found that 6.9% of the participants had PTSD symptoms at the baseline, with a decline in the follow-up waves (2.1%, 2.5%), whereas the prevalence of depression and general anxiety remained relatively stable throughout three time points (5%, 4.8%, and 5.6% for depression; 3.1%, 2.2%, and 1.8% for anxiety).

In a recent review by Newnham and colleagues [31], the findings presented a steady downward trajectory of PTSD after disaster events (23.8% at <6 months, 17.9% at 6–12 months, 17.75% at 12–24 months, and 11.35% at >24 months), but the prevalence of depression and anxiety remained elevated for years following the exposure; depression had a delayed (after one year) downward trajectory (27.5% at <6 months, 27.45% at 6–12 months, 28.60% at 12–24 months, and 13.5% at >24 months, and anxiety demonstrated a downward trajectory after 6 months (23.3% at <6 months, 22.23% at 6–12 months, 7.6% at 12–24 months, and 11.8% at >24 months). However, there are also studies indicating a reduction in depression over time [137,138] as well as an increase in depression between five and eight months after a disaster and one year later [139]. These variations in the results could be accounted for by the disaster types, exposure level, time elapsed since onset, or even population demographics [18,53].

Finally, although not common, there is evidence of the delayed onset of a mental problem where the symptoms do not develop immediately after the disaster but after some time [101]. The affected individuals with delayed psychological reactions tend to have high levels of symptoms immediately after the disaster, which may not be severe enough to fall under full diagnostic criteria, but, over time, the symptoms may become worse and develop into a full-blown mental disorder, e.g., PTSD [18]. For instance, Kessler and colleagues found that, following Hurricane Katrina [139], the PTSD rates increased from 15% to 20% between 6 and 18 months after the disaster. They observed that up to 25% of the people diagnosed with PTSD developed it at least 6 months after the disaster, termed delayed-onset PTSD [140,141].

### 3.2. Differential Psychopathology and Prevalence Rate

Note that, generally, the determinants of mental illness might vary by the type of illness [3]. Norris and colleagues suggested an explanation for this differential psychopathological outcome: the degree of disaster exposure plays a more significant role in PTSD development, whereas post-disaster factors (e.g., temporary housing) are more predictive of depression [142]. For example, a study on Hurricane Ike survivors found that PTSD was strongly predicted by the events experienced during and immediately after the disaster; however, depression was more a function of personal factors (e.g., low socioeconomic status) and life stressors [113]. Additionally, the wide range in prevalence rates across the studies may result from different categorizations of disorders (e.g., PTSS vs. PTSD), different cut-off points for disorders (e.g., depression—K6 score ≥ 10 vs. DASS score ≥ 4), inconsistent measurement techniques (e.g., screening test vs. diagnostic clinical interviews), disparate study designs (e.g., one-time cross-sectional vs. longitudinal), variations in timing for post-disaster assessment (e.g., 3 months vs. 12 months), selective recruitment (e.g., severely affected people vs. entire exposed community/population), and major reliance on convenience sample selection [18,27,53,116,143].

To summarize, typically, individuals facing adverse situations related to natural disasters resume normal functioning. However, some individuals of the affected population develop serious psychological problems associated with disaster exposure. The findings from disaster mental health studies have demonstrated both direct (e.g., length and intensity) and indirect (e.g., psychosocial factors) effects of disaster experience on mental health, that is, the onset of PTSD, depression, and anxiety.

Again, as noted above, following a natural disaster, generally, the affected individuals face several social and personal challenges, such as displacement, temporary housing, unemployment, and disruption in services and community resources. Thus, given the breadth and scope of the disaster, it is at least intuitively clear that the onset of a disaster event creates a “disaster period” that has a variable course and impact on the lives and well-being of the people within the affected community. Considering the life events that require a major adjustment to the routine life of an individual, a disaster can be counted as one of the important life events, or, to put it differently, an event of transition that significantly affects the lives, mental health, and well-being of the people who went through it. This is particularly important to evaluate because transition involves a process of life changes and adaptation to those changes that as a consequence produce a considerable amount of distress feeling [144,145,146]. Therefore, in the following section, we explain the possibility of disasters being important events of transition and the potential impact of this disaster-specific transition. Also, we propose a measurement instrument that can quantitatively assess the impact level of disaster-induced transition.

## 4. A Disaster as an Event of Transition—Measuring the Impact

When defining transition, Kobasa and colleagues said that transition is a period that requires adaptation, a process that is challenging but not necessarily stressful [147]. In contrast, Felner and colleagues defined it as a chain of events including multiple, potentially stressful, changes [148]. Later, Wilcox argued that transitions are a series of linked events; these chains of events include multiple loss events (e.g., a disaster leading to job loss, divorce, and financial difficulties), and they are likely to be stressful [149]. On the other hand, if these chains of events are made up of positive events or challenges that have been successfully resolved, they are more likely to produce a defense against stress [150]. Hobfall argued that stress is likely to be developed only when the loss is evident and the changes, transitions, and challenges are not stressful themselves [74]. However, according to Holmes and Rahe, any type of ‘change’ or transition that puts an end to one way of life and leads to another is often unsettling and distressful [144]. Based on this observation, it could be said that, following a disaster event, people go through a series of ‘changes’ (e.g., disruption in the normal routine, displacement from the house, or a business closure) in their life to which they have to adjust. The experience of this adjustment period could be an adverse one as the individuals might have to face a range of difficulties including damage to property, house renovation, dealing with insurance companies, etc. Therefore, these life changes, i.e., the transition from the pre-disaster to post-disaster situation, could produce distress, affecting people’s well-being.

One of the important transitional features of a disaster is that it appears to alter the affected people’s lives by replacing their old familiar life elements with new ones [151]. Simply put, at least during the onset of the disaster, the new life routines differ from the old ones, largely in their activities, places, people, and possessions. The post-disaster adjustment, however, can vary between individuals as some people return to their homes and jobs and engage in rebuilding, while others lose everything [152,153,154].

As discussed earlier, this adjustment phase could be emotionally taxing. Thus, in a broader sense, it can be said that disaster-induced transition can affect lives in two different ways [155]. First, it can change the way people live (i.e., material change); second, it can change how people feel (i.e., psychological change). In the following paragraph, we introduce an assessment tool that can measure transition-related material and psychological changes.

### Transitional Impact Scale

Although past disaster well-being research has considered several individual and social factors influencing the mental health of the affected people, the existing literature has been variable in linking those personal and social factors to the mental outcome following disasters [36,156]. One possible reason may be that there is large variation due to methodological differences across studies involving different assessment tools (e.g., from different quantitative rating scales to different qualitative interview questions) [53]. Thus, from a methodological perspective, introducing a uniform rating scale would make sense, which has the potential to both identify and quantify the aspects of a person’s life that have changed following a disaster event. In addition, the scale could empirically measure the relative magnitude of these different sorts of changes and could relate to the mental health consequences. For example, the Transitional Impact Scale (TIS, Table 1) has been designed to be short, psychometrically sound, applicable to a wide variety of theoretically important events, and assess the impact of the material and psychological changes regarding these events [155]. This 12-item scale was initially constructed to identify and characterize an event’s transitional properties, assess the nature of changes in the transition wrought, and measure the global impact of this event-specific transition [155]. Thus, the TIS can measure the personal, social, and environmental factors that have brought about changes in the lives of disaster-exposed people, having an impact on their mental health. The past research using the TIS demonstrated that, if an event scores higher than three (neutral), this indicates a moderate impact on life at least [157,158,159,160,161]. Therefore, using the TIS across disaster mental health studies could potentially increase the robustness and comparability of the disaster studies.

In contrast to the various assessment tools used in prior research, the TIS is advantageous in the disaster context because, by using this single rating tool, it is possible to identify the factors related to both individuals’ psychology (e.g., attitude and beliefs) and the society and environment they live in (e.g., people and places). In addition, the TIS provides an index of the magnitude of changes (i.e., psychological and material) and the degree of impact these factors have on the lives of people (e.g., mild to profound). These points indicate that, apparently, the TIS shares several features with the psychosocial framework that was discussed earlier. The TIS rating, particularly the material TIS, should be a robust predictor of mental health measures as people display adverse reactions to the negative consequences of their life-changing circumstances [161]. This notion is relatable to the stress literature. As noted above, the main finding to come out of this line of research is that life transitions or major transitional events often produce extreme stress [144,145,146,162,163]. This implies that the TIS can not only identify the quality and quantity of the changes the disaster brings about but also their relation to the mental well-being of the affected [164].

In summary, whether a disaster can be considered an important transitional event will depend on the level of direct, fundamental, and prolonged changes brought into the lives of the individuals who experienced it. Moreover, these disaster-associated life changes, i.e., transition, can be very stressful. In the section below, we explain how a disaster-specific transition can relate to mental health outcomes.

## 5. Relationship between the Transitional Impact of Disaster and Mental Health

From the transition perspective, we postulate that, in a broader sense, disaster mental health effects can be described in two stages—a *peri-disaster* stage and a *post-disaster* stage. Hence, individuals’ quality of experience with life changes during and after the disaster can influence their mental health and contribute to developing psychological problems [3].

In the *peri-disaster* period, that is, during and immediately after a natural disaster event, the experience of the disaster can range from direct to indirect. The direct experience with that disaster may be traumatic (e.g., escaping as the fire bears down, or almost drowning in the flood water) and as such can function as a source for PTSD or a PTSD-like reaction downstream. This notion is supported by prior research demonstrating relatively high rates of PTSD among disaster-affected individuals, at least during the first few months following the disaster event [14,103,104,105,106,107,108,110,111]. Of course, some people do avoid direct exposure to a disaster event and therefore do not experience it as a potentially traumatic event per se. The main point here is that greater or more intense disaster exposure may strongly predict a higher risk of developing mental issues, particularly PTSD-like manifestations. More specifically, during a disaster, individuals who experienced extreme stress or trauma, such as life threats, injury, and observing the death of significant others, will display a higher prevalence of mental problems compared to those who may have experienced those aspects to a lesser extent (e.g., having a loved one present at the disaster site) or those not having experienced them at all [101,165,166]. In comparison, the indirect experience of a disaster in this stage may constitute evacuation, temporary job displacement, disruptions in daily routine, etc. As mentioned before, this life disturbance brought about by a disaster usually leads to increased stress and gives rise to various mental health problems, such as depression, anxiety, and sometimes PTSD [20,167]. The prior evidence indicated that people who faced short-term job loss, temporary relocation, or were unable to take their belongings during an evacuation demonstrated a high prevalence of PTSD manifestation, depression, and anxiety [33,168,169]. Nonetheless, overall, the experiences at the outset of the disaster should be significantly distressful as the affected individuals’ emotional intensity is high [4].

Finally, the aftermath, or *post-disaster* period, can vary greatly from one individual to the next. The key predictor of the development and trajectory of disaster-related mental issues in this period is post-disaster life stressors [3]. Here, the ongoing stressors we can think about are different levels of property damage (from no loss to losing everything), impact on employment (temporary displacement to complete job loss), effect on social network and community infrastructure, etc. Hence, in this period, the disaster impacts the affected individuals in a more indirect manner. Some of those who were evacuated might return to their place, and, among them, some might be involved in prolonged and major rebuilding (e.g., a renovation or bank loan), whereas others might lose everything and have to restart their lives altogether (e.g., a new location or a new job). Assistance from government agencies and insurance companies may be available, but, during this period, it seems likely that the most important factor involves the nature and extent of the support provided by these agencies and the degree to which this support mitigates various long-term disaster consequences. Therefore, experiencing ongoing stressors in the aftermath of a disaster may influence the course of mental illness in the long term [170].

The key intuition here is that disasters can be devastating for some and not for others, and, when they are devasting, the misery can drag on for a long time and have a corrosive effect on the lives of the disaster-affected individuals, especially on their mental health. To elaborate, the degree of loss or damage of resources people experience following a disaster that led to the extensive rebuilding of lives in the aftermath drives the risk of developing mental health issues. The literature yielded similar findings [31,36,114]. Specifically, when people attempt to rebuild their lives after a disaster, they do so by housing reconstruction, returning/looking for jobs, and managing a social support system. This rebuilding or reconstruction may often last for years. While some accept their changes and losses, others develop resentment and anger, leading to an exacerbation of underlying mental distress [3,4,5]. Notably, the development of persistent negative psychological symptoms in the long-term mostly depends on the severity of the post-disaster experience and the post-disaster resource availability [5]. In Table 2, based on the study findings discussed earlier, some disaster-related factors that influence the mental health conditions of the affected people are listed. Note that this list is not exhaustive; rather, the purpose is to provide an overall idea of the types of factors that play an important role in developing mental problems over time.

**Figure 5:**
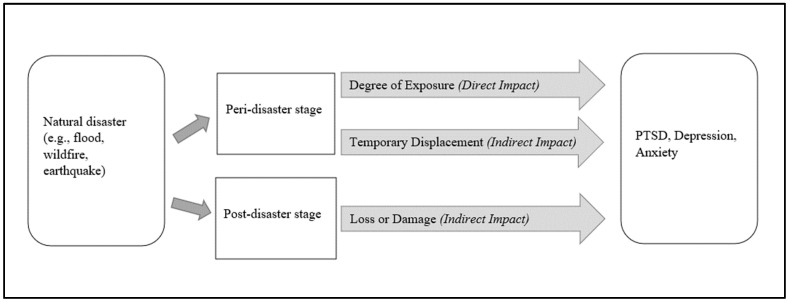
Variable impacts of disaster event on mental health.

In summary, the main points are the following: First, the same nominal disaster event can affect people very differently (Figure 5). Second, the long-term impact of a disaster on a person’s well-being is likely to reflect the aftermath or post-disaster stage.

As discussed earlier, from a transitional perspective, a disaster results in an abrupt halt in daily life and a marked change in the lives of every individual affected by it, at least at the outset, e.g., closure of workplace, educational institution, utility disruption, etc. [151]. It was also discussed that these sudden or new changes are related to negative mental health outcomes, especially for those whose lives are more affected than others, e.g., those displaced from their house [171,172,173,174,175]. Therefore, the magnitude of changes (material and psychological) involved in the peri-disaster and post-disaster stages can be measured empirically with the 12-item TIS, providing an index of the disaster event impact by assessing its transitional characteristics [155]. For example, it is reasonable to expect that people who experience more disaster-related changes, such as evacuation, in the peri-disaster period should, on average, produce an above mid-point material and psychological TIS rating compared to those whose lives were not affected to that extent.

Similarly, individuals who have experienced a devastating aftermath, that is, excessive loss or damage, and undergone forced involvement in the major rebuilding of their lives during the post-disaster period, should rate the material and psychological TIS items higher. As a consequence, parallel to the previously discussed disaster mental health findings, experiencing greater disaster-specific changes (i.e., higher material and psychological TIS ratings) should lead to an increased risk of developing adverse psychological conditions. More specifically, the long-term mental health consequences, namely PTSD, depression, and anxiety, should be influenced by the degree of loss/damage (i.e., change) and the level of life reconstruction people experience in the post-disaster period. Corroborating this notion, in a recent follow-up study of the 2013 Southern Alberta flood [176], we found that, after six years, depression and anxiety were highly related to the psychological TIS rating, and PTSD was strongly associated with both the material and psychological TIS ratings. We also found that, six years later, people who lost their jobs and houses as a result of the flood had a higher rate of depression and anxiety and possessed greater material and psychological TIS ratings than those who did not lose their jobs and houses. The implication here is to enhance our understanding of the differential aftermath or recovery process of a disaster, which could potentially assist in formulating need-specific intervention and policy development.

## 6. Strengths and Limitations

In terms of strengths, first, this narrative review illustrated a range of pathways by which disasters can affect mental health and well-being. Second, we proposed the notion of disasters being potentially transitional events and highlighted the importance of examining the critical link between the impact of a disaster transition and mental health. Finally, we introduced the TIS instrument, which can measure and compare the impact level of a disaster during the onset and aftermath and can possibly predict the long-term mental health consequences. Therefore, we believe that this review presents a novel approach to explaining the interconnection between natural disasters and mental health and well-being. This is particularly important because the connection between a disaster and its consequences on mental health is still far from being conclusively established. This issue is underlined by the intricacy of the current disaster mental health research. Moreover, this complexity is mostly because of the heterogeneity in what to assess and how to assess the mental health effect of a disaster [177]. Thus, in this context, the TIS can potentially be a valid measurement tool to address the ‘what’ and ‘how’.

In addition to the strengths, this review has some important limitations too. First, this was a narrative review and not a systematic one. In other words, a general literature search was conducted, the findings were summarized qualitatively, and an overview of the topic was provided instead of a systematic literature review. Second, the evidence searching and data retrieval were completed by only one researcher in a non-systematic way. That being said, the total number of studies, identical ones, and least-relevant studies obtained from the search were not considered during the data retrieval. Third, our literature search was limited to articles and reports published in the English language only and therefore likely missed relevant insights from studies published in other languages. Finally, while we structured our mental health consequences of a disaster from a transitional viewpoint, we acknowledge its limited capacity to delineate the true extent of the complex, interconnected social and psychological factors that affect mental health, and that there are other valid ways to interpret the impacts of a disaster on mental health. For example, recently, factors such as religiousness and spirituality garnered attention in well-being research and hence might play a role in disaster mental health effects, especially in post-disaster recovery [178]. Nonetheless, this narrative review contributes to providing a perceptive sketch of the transitional impact of a disaster on the mental health of those affected.

## 7. Conclusions

The current narrative review has confirmed the prior evidence that, through a range of direct and indirect means, natural disasters have a deleterious effect on people’s mental health. That being said, undoubtedly, a natural disaster is a distinctive event that in its breadth and scope affects individual lives to various degrees, permanently altering some to temporarily changing others. We already know that, typically, individuals who face significant adversity related to disasters bounce back and can resume normal functioning. However, for some, a disaster can produce short- or long-term psychological distress, leading to or amplifying mental illnesses, e.g., depression, anxiety, and PTSD. In other words, it is well known that there is an adverse long-term well-being impact of disasters as people’s initial and later reactions to a crisis event vary in degrees. Yet, less known are the transitional properties of disaster events and the ways they can affect the mental well-being of the exposed individuals during the pre- and post-disaster periods. Thus, an important contribution of this review is that it identified how a disaster event can change people’s lives (materially and psychologically), and the differential effect these changes can have on the mental health of the affected individuals. For instance, people who lost their jobs and houses due to a disaster may experience more disaster-related changes, i.e., transition, than those who did not experience, or experienced relatively fewer, changes (e.g., temporary relocation). As a consequence, it can be speculated that, the greater the disaster-related transition, the more adverse the mental health outcome.

On that account, it is important to quantitatively measure the disaster-induced transition, namely material and psychological changes, and to understand the differential mental health effects these changes can produce at the outset of the disaster and afterward. Hence, future research on the disaster mental health area can implement the TIS to numerically assess the transitional impact of a disaster and its relationship with mental health consequences during the pre- and post-disaster period. Using the TIS in this context can be beneficial in the sense that it can objectively determine the nature and intensity of the aftereffects of a disaster on people’s mental health, and, therefore, it can possibly enhance the generalizability of the research findings, another contribution of the current review.

Moreover, apart from individual consequences, further research should be conducted to determine the effects of disasters in community settings by comparing affected groups with non-affected or less-affected groups. These comparisons are of interest because, after a major disaster event, people share the experience and work together toward recovery [179,180]. Additionally, future research should consider cross-cultural and cross-regional differences while assessing the disaster’s mental health impact, for example, the annual flood in Bangladesh, a low-lying delta, vs. an incidental flood in the valley region, Western Germany. There should be a difference in the mental health consequences between those two groups as the resource availability and material and psychological consequences can vary widely in these two regions. This provides an impetus for implementing the TIS beyond the traditional disaster well-being impact assessment, particularly regarding the long-term impact. As the TIS can identify and measure disaster-related changes and their effect on mental well-being, using this uniform scale in cross-cultural disaster studies should increase the generalizability of disaster mental health consequences.

In conclusion, as was mentioned at the beginning, natural disasters are becoming more frequent, affecting a large proportion of the population. Therefore, it is imperative to follow the disaster trajectory after its onset, to assess the long-term mental health consequences, and to recognize that these consequences can be highly variable depending on the level of transition from the pre-disaster to post-disaster setting. Understanding the differential impact may well provide a framework for developing effective interventions for mental health and well-being, and bring the government, society members, policymakers, and key stakeholders together to be involved in disaster preparation, response, and recovery.

## Figures and Tables

**Table 1:** Transitional Impact Scale (TIS-12): 1 (completely disagree) to 5 (completely agree).

Item No.	Material Subscale
1	I spend my time in different places NOW than I did BEFORE the disaster event
2	I own different things NOW than I did BEFORE the disaster event
3	My material circumstances NOW are different than they were BEFORE the disaster event
4	The activities I engage in NOW are different from the ones I engaged in BEFORE the disaster event
5	The people I spend time with NOW are not the same people I spent time with BEFORE the disaster event
6	The place where I live NOW is different from the place I used to live in BEFORE the disaster event
	**Psychological Subscale**
7	My current attitudes NOW are different than the attitudes I held BEFORE the disaster event
8	I think about things differently NOW than I did BEFORE the disaster event
9	My emotional responses NOW are different than they were BEFORE the disaster event
10	My sense of self NOW is different than it was BEFORE the disaster event
11	My psychological state NOW is different than it was BEFORE the disaster event
12	My understanding of right and wrong NOW is different than it was BEFORE the disaster event

**Table 2:** Important factors for the development of mental illnesses during peri- and post-disaster periods.

Disaster Stage	Determinants of Mental Health Consequences, e.g., PTSD, Depression, and Anxiety
Peri-disaster	Direct: Exposure to danger, life threat, injury, death of significant ones, proximity to disaster, witnessing horrific scenes, family and friends at disaster site Indirect: Evacuation/temporary displacement, temporary unemployment, leaving belongings behind during evacuation
Post-disaster	Indirect: Home/property damage, home/property loss, job loss, forced relocation (permanent), disruption of community and healthcare resources, reduced/loss social support

Note. Poor socio-economic status, pre-existing mental illness, female gender, and being a young adult are common determinants across peri- and post-disaster stages.

## Data Availability

Not applicable.
